# Is resistance to Covid-19 vaccination a “problem”? A critical policy inquiry of vaccine mandates for healthcare workers

**DOI:** 10.3934/publichealth.2024035

**Published:** 2024-06-12

**Authors:** Claudia Chaufan, Natalie Hemsing

**Affiliations:** School of Health Policy and Management, York University, 4700 Keele St, Toronto, ON, M3J 1P3, Canada

**Keywords:** vaccine mandates, healthcare workers, critical policy analysis, COVID-19, medicalization and social control, bioethics

## Abstract

As the COVID-19 global vaccination campaign was launched in December of 2020, vaccination became mandatory for many healthcare workers (HCWs) worldwide. Large minorities resisted the policy, and the responses of authorities to this resistance led to damaged professional reputations, job losses, and suspension or termination of practice licenses. The joint effect of dismissals, early retirements, career changes, and vaccine injuries disabling some compliant HCWs from adequate performance has exacerbated existing crises within health systems. Nevertheless, leading health authorities have maintained that the benefits of a fully vaccinated healthcare labor force—believed to be protecting health systems, vulnerable patient populations, and even HCWs themselves—achieved through mandates, if necessary, outweigh its potential harms. Informed by critical policy and discourse traditions, we examine the expert literature on vaccine mandates for HCWs. We find that this literature neglects evidence that contradicts official claims about the safety and effectiveness of COVID-19 vaccines, dismisses the science supporting the contextual nature of microbial virulence, miscalculates patient and system-level harms of vaccination policies, and ignores or legitimizes the coercive elements built into their design. We discuss the implications of our findings for the sustainability of health systems, for patient care, and for the well-being of HCWs, and suggest directions for ethical clinical and policy practice.

## 1. Introduction

As the COVID-19 global vaccination campaign was launched in December of 2020, vaccination became mandatory for many healthcare workers (HCWs) worldwide [1]–[5]. The response was mixed, with many HCWs resisting the policy despite significant costs—loss of reputation, jobs, practice licenses, and personal relationships [6]–[9]. Evidence indicates that the joint effect of dismissals, early retirements, career changes, and vaccine injuries disabling some compliant HCWs from adequately performing has exacerbated existing crises in already overtaxed health systems [10]–[16]. Nevertheless, leading health authorities have maintained that the benefits of a fully vaccinated healthcare labor force—believed to be protecting health systems, vulnerable patient populations, and even HCWs themselves—achieved through mandates, if necessary, outweigh potential harms [17]–[19].

Informed by the tradition of critical policy and discourse studies, especially the framework proposed by Carol Bacchi called “What is the problem represented to be” (WPR), we examine the expert literature on COVID-19 mandated vaccination for HCWs. We find that this literature neglects medical evidence contradicting the official claims about the safety and effectiveness of vaccination, dismisses decades-old scientific knowledge about the contextual nature of the virulence of microbial agents, miscalculates the impact of mandated vaccination policies on patient care and on the sustainability of health systems, and ignores or legitimizes the coercive elements built into their design. We first provide a background discussing selected aspects of the epidemiology and pathophysiology of COVID-19 to enable readers to better appraise our study and overall thesis. Next, we describe the theoretical and methodological approaches that inform it. This section is followed by our findings and analysis and, subsequently, by a discussion of our study and its limitations in the context of the broader literature. We conclude by elaborating on our study's implications for the sustainability of health systems, the quality of patient care, the well-being of HCWs, and ethical clinical and policy practice. This study is the first arm of a project appraising the impact of the COVID-19 policy response on HCWs and on health systems (Open Science Frame Registration, https://osf.io/z5tkp), which is part of a larger project examining geopolitics, medicalization, and social control in the COVID-19 era.

## 2. Background

In October of 2020, an article in *The Lancet* entitled “Scientific consensus on the COVID-19 pandemic” laid out in no uncertain terms what an appropriate COVID-19 policy response should look like: the author asserted that COVID-19 had a lethality “several-fold higher than the seasonal flu” that anyone, regardless of age or clinical background, could experience severe disease and death, that there was little reason to rely on natural immunity, and that mass masking, lockdowns, rapid testing, contact tracing, and isolation were critical to control viral transmission “until safe and effective vaccines and therapeutics arrive” with anything less being a “dangerous fallacy unsupported by scientific evidence” [20] (pgs. e71–e72). To this day, the “scientific consensus” remains largely unchallenged. The World Health Organization (WHO) has added to it the issue of “vaccine equity” asserting that despite public perceptions to the contrary, “COVID-19 is not over” and that “everyone, everywhere, should have access to COVID-19 vaccines” including HCWs, who are deemed a “high-priority group” [21].

Over time, however, facts accumulated that called into question this ostensible consensus: as early as the spring of 2021, it became clear that infections were occurring among the vaccinated, dubbed “breakthrough infections.” For example, the US Centers for Disease Control and Prevention (CDC) communicated that by April of that year, 46 states had voluntarily reported 10,262 breakthrough infections, and that beginning in May, only those leading to hospitalization would be tracked [22]. As of then, whenever tracked, about 75% of cases would continue to occur among fully vaccinated populations [23]. Also, research published in the *European Journal of Epidemiology* of 68 countries and 2947 US counties showed no correlation between COVID-19 vaccination rates and cases [24], thus challenging claims that vaccination prevented transmission, hospitalization, or death. This finding was unsurprising: already in 2020 leading scientists had noted that the original trials of at least one of the most acclaimed products, the Pfizer–BioNTech COVID-19 vaccine, never included transmission, hospitalization, or death as clinical endpoints [25],[26], which indicated that, ethics aside, there never was a rational justification for mandates.

Also, early on, it was noted that a positive PCR test—intended “for research [and not] diagnostic procedures” need not indicate clinical illness, and that in “an asymptomatic individual without known close contact with an infectious individual, especially in a low prevalence setting, the finding of a positive RT-PCR test result should raise the possibility that the result is a false positive” [27] (pg. e160). It followed that using high numbers of “cases”—indicated by positive PCR tests—to gather support for public health measures (see for instance [28],[29]) was not justified. Also early on, a study of close to 10,000,000 individuals in Wuhan, China—“Ground Zero” for COVID-19—revealed no positive tests among 1174 close contacts of asymptomatic cases [30], calling into question the claim by leading health authorities that persons with no symptoms yet a positive PCR test threatened the public's health and should be isolated [31],[32]. Also early on, an article in *The Lancet* found that during the so-called Delta wave, “fully vaccinated individuals [had] a peak viral load (25%), similar to unvaccinated cases (23%), and [could] efficiently transmit infection in household settings, including to fully vaccinated contacts” [33], challenging official claims that vaccination halted or reduced transmission, claims offered as rationale for mandated vaccination [34],[35].

Over four years into the onset of the COVID-19 crisis, it has been well established that risks of poor health outcomes are not distributed equally: the infection fatality rate starts as low as 0.0003% (near zero) among children and adolescents and increases to 0.5% in those aged 60–69 [36], and persons with comorbidities, as expected, are at significantly higher risk of severe outcomes than those without them [37]–[39]. Further, the much-publicized “95% vaccine effectiveness” that led many among the unsuspecting public to believe that the vaccine was successful in preventing illness 95 out of 100 times referred to *relative*, not absolute, risk reduction, meaning that *if* exposed, *and* infected, *and* only upon developing the COVID-19 disease, vaccines afforded a protection of *minor illness of under 1%*, not counting risks of harm [40]. It is also well established that exposure does not always lead to infection [41], that antibody levels may not predict disease severity [42], that natural immunity is durable, comprehensive, and strong, including among HCWs and compared to a faster waning vaccination immunity [43]–[46], and that early treatment with repurposed drugs has reduced severe illness, hospitalization, and death [47]–[50].

As well, a study of over 50,000 employees from the Cleveland Clinic in the USA led to the “unexpected finding” that *more* boosting correlates with *higher*, not *lower*, risk of infection [51] (pg. 7), and documents released under court order in August of 2022 have revealed about 1.6 million adverse events that had been kept hidden from the public [52]. Even leading health agencies have now abandoned claims that COVID-19 vaccines stop viral spread [53], an admission that further calls into question mandated vaccination. Indeed, the very authorities repeatedly reassuring the public that vaccines were safe have admitted to their harms. For example, a December 2023 UK government report admitted to “limited experience with use of the COVID-19 mRNA Vaccine BNT162b2 in pregnant women” and to adverse events post-vaccination, including hypersensitivity, anaphylaxis, and myopericarditis [54]. Indeed, at the time of this writing, there remains little doubt that COVID-19 vaccines are associated with a wide range of serious adverse effects. These include an observed versus expected (OE) ratio for acute disseminated encephalomyelitis of 3.78, for cerebral venous sinus thrombosis of 3.23, and for Guillain-Barré syndrome of 2.49 [55], and an excess risk of serious adverse events of special interest, including death, between 10.1 and 15.1 [56]. As a result, two medical societies have called for a worldwide withdrawal of COVID-19 vaccines from the market, on scientific and ethical grounds [57],[58]. Finally, the empirical basis of the rationale to mandate vaccination among HCWs to protect vulnerable patients has been challenged by accumulating evidence. By way of example, a study of NHS elderly care home staff in England indicated that while mandates did reduce the rates of unvaccinated HCWs—unsurprisingly given that refusal was punished with job loss—they also led to an important—between 3 and 4%—reduction in the work force, equivalent to 14,000 to 19,000 fewer HCWs, with negative impact on the health and well-being of residents in these establishments, thus undermining the very goal of the policy [59].

This brief account, along with the work and public declarations of many dissenting scientists and practicing physicians worldwide [50],[60]–[62], should call into question that there ever was a “consensus”—scientific, policy, political, or ethical—on what the public health response to COVID-19 should be. Thus, the goal of our research: to “problematize” the dominant “problem representation,” the “problem” of unruly HCWs, as we explain in the following section.

## 3. Methods and data

As mentioned earlier, our study is grounded in the critical policy studies tradition, especially the work of Carol Bacchi, who proposed a conceptual framework guided by the question “What is the problem represented to be” (hereafter WPR) to inform research on social issues of potential policy relevance [63]. What distinguishes the WPR from other frameworks is that it does not *assume* the representations of social problems held by official institutions or persons, hereafter “dominant representations”, to subsequently discuss which interventions “work”. Instead, the WPR approach proposes a series of questions to assist with identifying dominant representations, by examining the policies proposed to tackle said problems. In other words, what Bacchi proposes is not to identify policy “problems” but to “problematize” dominant problem representations by scrutinizing assumptions and underlying power dynamics [63] (pg. 30). Guided by this framework, we crafted the following research questions: 1) What is the problem represented to be in the expert literature concerning the policy of vaccine mandates for HCWs, and what assumptions inform it? 2) What is left unproblematic in this problem representation? and 3) How could this representation be challenged?

We also drew from other traditions that share a concern with the role of power in organizing social affairs. Such is the call to “study up”, proposed by anthropologist Laura Nader, who invited researchers to study power and domination not only from the perspective of the powerless but also of the powerful [64]. Nader's perspective informed our choice of socially recognized expert, i.e., peer-reviewed literature, as a point of entry to appraise mandated vaccination policies for HCWs, and of documents by leading national and international health authorities like the CDC and the WHO as representing the official consensus on COVID-19 policy. Similarly, the notions of “ingroup/outgroup,” aspects of the sociology of stigma, informed our analysis of how contingent social categories—nationality, ethnicity, political ideology, behaviors, and so on—are deployed to indicate that someone does not belong to the dominant group, the “Us” [65]–[67]. These notions also help to explain how, when the outgroup comes to be perceived as threatening the economic, political, social, and/or moral order, it can become permissible, and even morally imperative, to “target [it] for discrimination, prejudice, hostility and even violence” [68] (pg. 6). Finally, we drew from “critical discourse analysis”, which studies how diverse forms of communication reproduce or challenge relations of domination [69], “document analysis”, which considers documents as “social facts” able to capture and convey meaning [70], and thematic analysis, which invites researchers to identify salient messages in the data [71]. Informed by these traditions, we examined a systematic selection of refereed journal articles that discussed vaccination mandates among HCWs to identify the dominant “problem representation”, discursive strategies, and conceptual and ethical tensions within this research area. Given our joint background and work in the social sciences, humanities, health policy, and medicine, we also relied heavily on COVID-19 epidemiology, immunology, and pathophysiology to interpret our observations.

Throughout this paper, we use the terms “vaccine” and “vaccination” to refer to any of the products labeled as vaccines by the WHO as of December 2023 [72], noting that those using the mRNA platform (e.g., BNT162b2 or mRNA-1273) fall under the definition of “gene therapy” products (GTPs) [73],[74]. Irrespective of the technology, we argue that the term “vaccine” is relevant to our investigation in at least two ways: one is legal, in that the act of calling a product a “vaccine” affords drug companies producing them unique liability protections not enjoyed by other products [75],[76]; the second one is sociological, in that the term “COVID-19 vaccine” elicits the social trust afforded, deservedly or not, to “traditional” vaccines. Also, when discussing vaccination/vaccines, we refer specifically to *COVID-19* vaccination/vaccines, and when discussing mandates, we refer to *COVID-19* vaccination mandated as a condition of employment, unless otherwise specified. Finally, when discussing the use of “misinformation” in the body of data—defined by the U.S. Surgeon General as “information that is false, inaccurate, or misleading according to the best available evidence at the time” [77] (pg. 4)—we make no effort to evaluate if authors distinguish it from “disinformation” that, as per the National Library of Medicine, indicates *intention* to spread false information [78]. We also assume that authors imply “mis-disinformation” when they refer to “conspiracy theorists”. This is because “conspiracy theory”, meaning false or misleading, as well as implausible and irrational, information, is how the concept is defined by alleged experts in the topic (see for instance [79],[80]).

**Figure 1.**
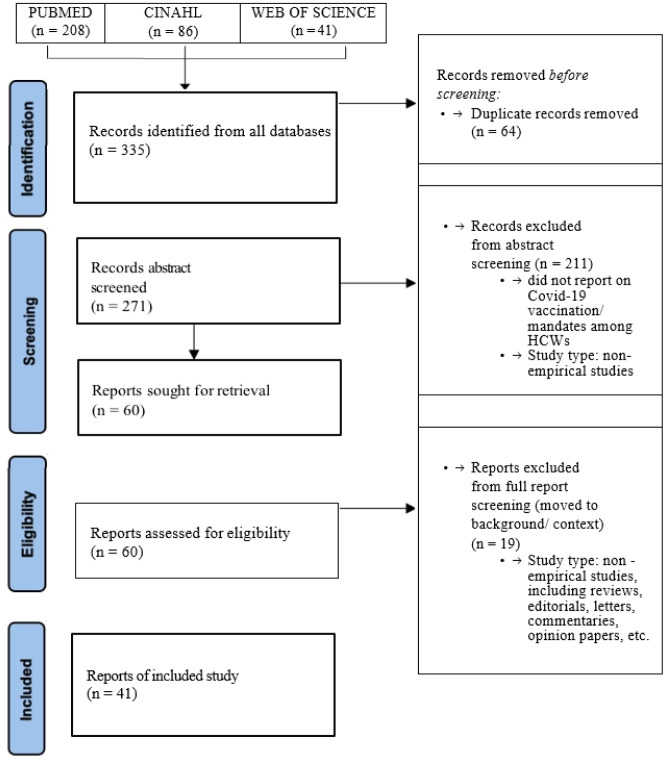
Flow chart of study selection.

On October 13, 2023, we searched PubMed, Web of Science, and CINAHL for articles on COVID-19 vaccines and mandates for HCWs. For the PubMed search, we combined the medical subject heading (MeSH) terms “health personnel”, “COVID-19 vaccines”, and “mandate” or “policy”. For the other searches, we replaced “health personnel” with “healthcare workers”, as the latter term returned more articles than the former. The PubMed search identified 208 articles, the Web of Science search 41, and the CINAHL search 86 that we screened for relevance to the topic of vaccine mandates or vaccination policies for HCWs, restricting our selection to empirical studies. Our search resulted in 22 PubMed, 7 Web of Science, and 12 CINAHL studies, a total of 41 studies included for analysis (Figure 1). We read the articles selected in their entirety, and coded them guided by our research questions, noting salient themes within them, illustrating our interpretations with quotations, and resolving discrepancies through discussion. Because our data were publicly available documents, no IRB approval was required. In the following section, we describe demographic and other characteristics of the data, followed by a section with our findings and WPR analysis of the most salient themes, organized around our research questions.

## 4. Findings

Our search did not include geographical or temporal restrictions, but we only identified records published between 2020 and 2023 because one inclusion criterion was an exclusive focus on COVID-19 vaccine mandates. The 41 included empirical studies were methodologically diverse and comprised quantitative observational studies (N: 25/41, 61%); qualitative studies (N: 9/41, 22%); and mixed methods studies (N: 7/41, 17%). Studies were also conducted in diverse locations including the United States of America [USA] (N: 19/41, 46%); Canada (N: 4/41, 10%); the United Kingdom [UK] (N: 3/41, 7%); Australia, Belgium, Switzerland, and Nigeria (N: 2/41, 5%); and Oman, Czech Republic, Finland, Greece, India, Poland, and Pan-European (N: 1/41, 2%). The first authors of the reviews were affiliated with institutions across 15 countries, with the USA exhibiting the highest (N: 20/41; 49%) number of author affiliations. Funding sources were declared in most studies (N: 32/ 41, 78%), with most among them (N: 22/32; 69%) relying on funding. Most studies (N: 38/41; 93%) provided a conflict-of-interest declaration, with most among these reporting none (N: 31/38, 82%). Among those that reported conflicts of interest (N: 7/38, 18%), examples included funding or consulting fees from COVID-19 vaccine manufacturers such as Pfizer, Moderna, Johnson & Johnson; being a board member for a vaccine manufacturer such as MSD European vaccine, AstraZeneca; receiving financial support from the Vaccine Confidence Project, housed at the London School of Hygiene and Tropical Medicine and whose mission includes to “better understand growing vaccine scepticism around the world” [81]; and serving on the Speaker Bureau for Pfizer (Tables 1 and 2).

Populations of HCWs were diverse, with the most studied among them being low-income workers, such as home care and nursing home employees (N: 9/41, 22%); all employees of a particular medical department, hospital, or health system (N: 8/41, 19%); HCWs of all professions, including non-clinical and support staff (N: 6/41, 19%); and patient care providers including select multiple groups (e.g., physicians, nurses, EMTs) (N: 5/41, 12%). Examples of other study populations, captured by more than one study, included: healthcare providers (medical, allied health) and administrators (N: 3/41, 7%); nurses (N: 3/41, 7%); students in nursing and midwifery (N: 2/41, 5%); and medical students and healthcare providers (N: 2/41, 5%). The remaining populations were unique to each study, including Emergency Medical Service workers (N: 1/41, 2%); HCWs providing patient care and laboratory services (N: 1/41, 2%); and healthcare chaplains (N: 1/41, 2%) (Table 3).

### 4.1. What is the problem represented to be in the expert literature concerning the policy of vaccine mandates for HCWs and what assumptions inform it?

As per the WPR approach, we inferred the answer to the question of problem representation from the courses of action, namely, the policies proposed by the dominant messaging we identified in the data. The thrust of it was that it was imperative to persuade, manage expressions of resistance and dissent, and if necessary, coerce reluctant HCWs to get vaccinated, given their “suboptimal” vaccine uptake— “suboptimal” even when the reported uptake was acknowledged to be significantly higher among them than among the general population [82]. It followed that the “problem” was represented to be a minority of HCWs who, for reasons that study authors perceived as unjustified, were not readily disposed to comply with what was, and continues to be, held by health authorities as the only scientifically sound and morally justifiable public health measure able to protect health systems, patients, and communities, and even HCWs themselves from an alleged, unprecedented threat, namely, infection by SARS-CoV-2 and its repercussions.

Consistent with the logic of this representation, the theme of “suboptimal uptake”, pervasive across studies, meant *anything less than universal uptake*. For example, Achat et al. identified “exceptionally high” vaccination acceptance among HCWs—an observation confirmed by Shaw et al., who found a vaccination rate among HCWs of 87.7% [83], and by a survey of nurses in Europe that found coverage rates “well above the 70% target threshold advocated by WHO” [84] (pg. 133)—while at the same time discussing “suboptimal” vaccination rates [85] (pg. 169). So why the concern? Because, as noted by Tylec et al., HCWs “cover the front line in the battle against infectious diseases” [86] (pg. 237) and, according to Hubble et al., may “act as a vector and transmit the disease to susceptible patients, coworkers, friends, and family [so] it is essential that all [of them] be vaccinated against COVID-19” [87] (pg. 571). Further, Thaivalappil et al. asserted the importance of HCWs overcoming “hesitancy” because they “are often the preferred source of information for patients [...] and widespread vaccine acceptance among providers may enhance vaccination coverage within the public” [88] (pg. 696). This position was shared by Heyerdal et al., who wrote that HCWs “are the most influential sources of laypersons' vaccine decisions” [89] (pg. 890). Lucia et al. extended this role to *future* HCWs, i.e., students, because they *“*will be entrusted with counseling vaccine-hesitant patients,” so their projecting confidence was “critical for presenting a unified message of strong vaccination support from the medical community” [90] (pg. 448).

As revealed by our analysis, the dominant problem representation hinged on at least three assumptions: first, that unvaccinated, unlike vaccinated, HCWs (or persons in general), represented a health threat to the vulnerable, primarily patients in healthcare settings; second, that COVID-19 vaccines are safe and effective, and that whatever the risks of mass inoculating HCWs across place, time, demographic characteristics, or clinical background, even if occasionally admitted to exist, were always outweighed by the alleged benefits of the practice; and third, that concerns about vaccine harms or proposed alternatives to vaccination were grounded on “misinformation”. Therefore, across the data, authors emphasized that HCWs should be vaccinated to protect “susceptible patients, coworkers, friends, and family” implying that once vaccinated, they would no longer pose a threat [87] (pg. 571), and that the safety concerns of these HCWs were unsubstantiated due to “the proven efficacy and safety of COVID-19 vaccines” [91] (pg. 442), because these products had “undergone randomised clinical studies to ensure they are of high quality, safe, and effective” [92] (pg. 2). Another author reported on one HCW, who experienced vaccine injuries that led them to not pursue further doses, yet whose concerns had been dismissed by colleagues [89]. Other authors discussed the role of natural immunity in vaccination decisions and even acknowledged that unvaccinated HCWs were more likely to have recovered from infection [93]–[95], yet dismissed this protective effect because the CDC recommended vaccination “regardless of previous SARS-CoV-2 infection” [95] (pg. 5).

But readers may wonder, if full vaccination is the only evidence-based *and* morally acceptable option for HCWs, why would some of them, despite their medical training, hesitate or reject it? Several explanations, framed as driven by “misinformation” and “conspiratorial thinking” were offered by authors, including unsubstantiated concerns with vaccine harms, underestimation of the threat posed by COVID-19, undue trust in natural immunity, or “politicization of the COVID-19 pandemic” [87],[96]–[99]. By way of example, having dismissed fertility concerns with vaccination, Hoffman et al. proffered that “it would be valuable for [HCWs] to counter misinformation [...] including concerns about vaccine safety while pregnant or breastfeeding” [100] (pg. 756), while Shaw et al. recommended that “misperceptions, such as lack of research data, rushed vaccine development, and misinformation about the vaccine's impact on fertility and reproduction” be corrected through “education” [83] (pg. e820).

Some authors acknowledged that racialized HCWs—“racialized” is recommended over “visible minorities” by the Canadian Human Rights Commissions [101]—were less accepting of COVID-19 vaccines [93],[95],[102]. However, while this “mistrust” was deemed excusable on grounds of its roots in negative, historical or current, experiences with medical institutions [103],[104], it was still presented as misinformed, i.e., not grounded on *real* evidence of harm but rather a misapplication of this mistrust in the COVID-19 case. It was also presented as problematic given its potential to “exacerbate racial/ethnic disparities in COVID-19 outcomes” when these HCWs served patient populations in their own communities [102] (pg. 286). For example, in their study of HCWs in Nigeria, Iwu et al. stated that: “despite their knowledge and training [vaccine hesitant] HCWs continue to be at risk of being trapped within the cycle of misinformation and vaccination fear” [99] (pg. 7).

An interesting variant of the theme of acknowledging the perspective of HCWs who failed to wholeheartedly embrace the dominant narrative was the case of authors who recognized that vaccination decisions are complex and influenced by many factors yet disqualified this complexity in the very act of acknowledging it, attributing it in the last instance to psychological attributes of “deviant” HCWs. For instance, a study of Emergency Medical Services personnel found that approximately 50% of unvaccinated HCWs offered as primary reasons “concerns about safety, effectiveness, side effects, [fear of] acquiring COVID-19 illness from the vaccine itself, and general antivaccine sentiment”, i.e., clearly a complex interaction of factors as per the authors' own description, who nevertheless reduced this complexity to “misinformation”, because even if “the rationale among the non-vaccinated is complicated [...] misconceptions [about safety] prevail” [87] (pg. 575).

Several authors went a step further and framed vaccine concerns as not only driven by “misinformation” but by “conspiracy theories”—meaning not only *false* but also *paranoid* beliefs about the motives of vaccine promoters or the harms of vaccination—in which case the HCWs in question were framed not only as ignorant but also as having crossed the boundaries of acceptable moral behavior *qua* HCWs *and* members of civil society, thus deserved little empathy, and even punishment. For example, one author elaborated on how health organizations could dismiss exemption requests by weeding out applicants who were misinformed or believed in “conspiracy theories”, such as “a person espousing a view that COVID-19 vaccinations contain a 5G chip or are being used to create a new world order” [105] (pg.144). Authors of another study of chaplains involved in evaluating religious exemption requests reported that “featuring prominently in comments from chaplains was the difficulty navigating requests in the context of anti-science, anti-vaccine, and politically charged public discourse” and “dismay over staff's reliance on inaccurate information and use of the religious accommodation policy to skirt the vaccine mandate for political reasons” [103] (pg. 7). In another study, of Australian nursing and midwifery students, authors reported that participants expressed outrage that their fellow students would not support COVID-19 vaccination due to their beliefs in conspiracies, expressing that “healthcare students who do not understand the importance of vaccines in pandemics and for public health in general should not be in the healthcare industry” and that “there should be very little tolerance for nurses and student nurses buying into conspiracy theories when we have access to a HUGE body of peer reviewed evidence” [92] (pg. 5). The authors appeared to excuse the expressions of intolerance of study participants, as gleaned by their conclusion that “COVID-19 remains a global health risk and therefore further research is needed of vaccine acceptance amongst the future health workforces” [92] (pg. 6). Given this problem representation, achieving universal vaccination among HCWs by any means necessary—ideally through education, persuasion, and provision of “accurate” information, or through mandates if necessary—was deemed the only solution. Hence one author stated “COVID-19 vaccine mandates for [HCWs] may ultimately be the only viable strategy for achieving an adequate level of vaccination among health care workers”, especially for HCWs who were “on the fence” yet presented “modifiable barriers to vaccination”—such as HCWs waiting for more long-term safety data [102] (pg. 294). The call for mandatory vaccinations for HCWs cut across continents, with studies from low- or middle-income countries also recommending it given concerns over “vaccine hesitancy” [99],[106]–[108] and the health challenge “hesitating” would unleash.

However, the call for mandates was not always blunt, and some studies, upon identifying significant resistance among HCWs rank and files recommended them “as a tool of last resort” [109] (pg. 12). Many authors even warned that mandates may be “unwarranted” given high uptake rates or significant buy-in identified among HCWs [13],[85],[102]. Instead of mandates, these authors recommended “targeted interventions addressing apprehensions” [85] (pg. 96), or “education” to persuade HCWs found to be open to accepting vaccination in the future [110]. Other authors argued that “the dichotomous ‘anti-vaccine vs. pro-vaccine’ separation of HCWs may not be adequate in informing interventions”, and advocated for *targeted* interventions depending on whether HCWs were “hesitant”, “unconcerned”, and so on [111]. Further, since “misinformation” framed as a major barrier to full societal recovery, was seen as resulting from damaged psyches prone to faulty beliefs, authors recommended interventions informed by the behavioral sciences, while those identifying “hesitancy” among racialized communities also called for rebuilding their trust by recruiting “influencers” in these communities to encourage vaccination [102].

Finally, another salient theme was the perceived need to protect healthcare systems from the threat posed by unvaccinated HCWs, along with the confidence that mandated vaccination for HCWs could cause no harm. So, for example, in a study of U.S. nursing homes, authors offered as evidence of no harm to patient care and health systems (the focus of their study) that the “federal COVID-19 vaccine mandate has not caused clinically material changes in nursing home's nurse aide and licensed nurse staffing levels”, even as their data showed, and authors admitted, that mandates were “(at most) modestly associated with changes in staffing levels, regardless of state mandate status” in a *downward* direction [112] (pg. 451). An ancillary concern was the need to maintain social order within the healthcare labor force, a need that demanded a continuing emotional investment on the part of those HCWs who were less than enthusiastic about vaccination. As Heyerdahl et al. noted, to ensure full affiliation with the dominant group required *not only conforming* to social and institutional pressures to be vaccinated *but also concealing any vaccine related concerns*, a situation described by the authors as “unspoken vaccine hesitancy”, because “merely voicing vaccine-related concerns entail [ed] a risk of being lectured, mocked, stigmatized, or labeled as conspiracy theorists and ‘anti-vaxxers'” [113] (pg. 1).

In sum, the conviction, or at least hope, appeared unwavering that the proposed remedy, universal vaccination for HCWs, through mandates if necessary, could never cause more harm than the “disease” it was meant to cure, namely, HCWs' less than full embrace of COVID-19 vaccination. It is to the assessment of these harms that we now turn.

### 4.2. What is left unproblematic by the dominant problem representation?

Conviction and hope of no harm notwithstanding, the unprecedented professional and personal—social, financial, emotional, and physical—impacts of the policy of mandated vaccination were apparent across the body of data, even if left unproblematic, generally dismissed as “collateral damage” of a once-in-a-century “war” against a lethal virus, and always amenable to “policy fixes”. For instance, in a Canadian study, most HCWs described “a pro-vaccine social circle” that made the expression of dissenting opinions exceedingly difficult [88], whereas two UK studies also reported a strong social norm in the workplace—“workplace pressure”—to be vaccinated [104],[114], which in the end compelled reluctant respondents to get vaccinated against their will. Similarly, interview and focus group participants in a Belgian study reported “strategic silences”, meaning avoidance by HCWs of the topic of vaccination to prevent conflict with co-workers and maintain employment [89]. Even studies qualifying dissenting HCWs as “misinformed” appeared to be cognizant of the negative impacts of vaccine mandates on them, on patient care, and on health systems, noting that mandated vaccination risked “completely isolating [unvaccinated HCWs] and losing them to the profession” at a time of staff shortages [111] (pg. 12). According to the authors, however, this negative impact could be offset, at least in part by, for example, recommending that employers “encourage or mandate that vaccine boosters [be] taken on days of, or immediately preceding, planned time off (such as weekends or vacation)” to improve productivity [115] (pg. 3179).

In addition to the “unproblematized” nature of the multiple harms caused to HCWs by mandated vaccination, the harms to health systems did not go unnoticed. So, for example, one study of vaccine mandates in a U.S. nursing home facility presented them as successful, as they had achieved “100% compliance”, seemingly well worth the loss of 18 HCWs that the authors noted had resigned, and of seven who were exempted or on leave of absence [116]. Another study reported “minimal impact” of mandates on staffing in a U.S. radiology department, noting that by anticipating potential disruptions in patient care resulting from mandated vaccination, some establishments were recruiting “nurses from the Philippines to fill the health care work gap” [91] (pg. 444). Similarly, a study from British Columbia, Canada, found that upon introducing a vaccine mandate, 6.4% of HCWs in a rural location remained unvaccinated compared to 3.5% of urban HCWs, “despite consequent employment termination” [117] (pg. 55). The authors subsequently concluded that while “the policy fell short of achieving high levels of uptake”—presumably, vaccination rates of 93.6% and 96.5%, respectively, *were not high enough*—increasing uptake remained critical, and that perhaps “community-based strategies” and “trusted local leaders” should be explored, with attention paid to not “aggravating staff shortages”, especially in rural settings [117] (pgs. 56–57). However, the authors failed to explain how staff shortages could be prevented *with the same policy that had led to them*, even as they acknowledged potential “downsides” of mandates, such as the staff shortages they themselves acknowledged, with “long-term effects not yet known” [117] (pg. 54). Still, other authors seemed undeterred, and recommended mandates regardless of their staffing impacts. For instance, a study of nursing homes reported that vaccine mandates with a test exemption had proven largely ineffective to increase vaccine uptake [118]. To address this perceived problem, the authors recommended policies such as not offering test-out options to new staff and to mandate vaccination “to [HCWs] in other settings and in other low-wage jobs, thereby limiting the option for unvaccinated staff to work elsewhere” [118] (pg. 765).

Finally, the coercive nature of the practice, while admitted by at least some authors, was *normalized*, i.e., presented in a “this is the way the world is” fashion, if at all mentioned. For instance, one study noted, in a matter of fact way and with no further elaboration, that “some [HCWs] in our study complied with the immunization procedure, not because they were convinced of its benefits, but rather due to pressure from the government's suspension of work penalties” [109] (pg. 12). Another study of Australian nursing and midwifery students reported that at least some students felt coerced or forced by vaccine mandates and thought that the policy was unfair [92], once again with no elaboration by study authors. A study of nursing home staff caring for people with disabilities in Switzerland found that vaccination decisions were described by participants as “taxing”, and appeared to be informed by multiple factors, including “fear of discrimination” against those unsure or opposed to vaccination. However, rather than concluding that perhaps the policy of mandated vaccination was misguided, the authors discussed how, to “encourage vaccination” employers and authorities should provide “quality information” ensure the “presence of trustworthy interlocutors” and in general “promote critical thinking and science literacy to mitigate common barriers to informed and self-determined decision-making” [119] (pg. 100181), thus *psychologizing* the reasons for participants' concerns.

The well-documented emotional distress caused by the policy notwithstanding, some authors remained convinced that it was still worth enforcing, for the greater good and that of dissenters themselves. Thus Lee et al. proffered that mandating vaccination for HCWs was more “equitable” than not mandating it, because their analysis of U.S. national survey data had revealed *higher* vaccine uptake among HCWs in facilities with mandates, and *higher* rates of refusal among younger HCWs, with high school or less education, living below the poverty level, and uninsured, concluding that the most *vulnerable* HCWs would be the *most* benefitted by mandates [120]. The authors' reasoning appeared to be that while correlation does not imply causation, the higher rates of refusal of vaccination among socially disadvantaged HCWs indicated that they would be the most “benefited” by mandates, shown to increase uptake [120] (pg. 7481), supporting “vaccine equity,” even if imposed through coercion.

### 4.3. How could the dominant problem representation be challenged?

Despite the remarkably homogeneous problem representation, regardless of author affiliation with high-, middle-, or low- income countries, disciplinary background, and study population or design, and across the three years captured by our data, the resistance of HCWs to the policy of mandates was apparent, even among HCWs who, for whatever reason, had complied with it. For example, one study of Australian nursing and midwifery students found that some participants were both “anti-mandate” and “pro-vaccination” [92], indicating that having been vaccinated, even if willingly, did not indicate support for mandates. Another study of Emergency Medical Services professionals found that only 18.7% supported mandates [87]. Another one reported that when consulted prior to the introduction of a vaccine mandate, nursing home HCWs had replied that mandates “sent the wrong message about vaccine safety and coercion” [116] (pg. 1999). Yet another recommended “multipronged approaches [that] shift away from coercion and punishments” [88] (pg. 703), while another one found that even senior healthcare management “emphasized the importance of personal choice and anticipated push-back to mandatory vaccination” [104] (pg. 1566). Indeed, in one study showing high support (62%) for mandates among HCWs, many participants opposed them if the consequence was job termination, with one participant noting that “leveraging people's jobs in order to get them to get a booster isn't the way to do this. You are just creating a staffing shortage that could be avoided”, and another one stating that “I am fully vaccinated but I resent the mandate on boosters—especially termination for noncompliance” [121] (pg. e2144048–5).

Finally, one study exploring attitudes of care home employees in England identified strong opposition to vaccine mandates, with most participants stating that they would rather quit than be vaccinated if vaccination was a condition of employment [114]. In other studies, participants questioned mandates on the grounds that they had cared for clients before a vaccine was available. If they had been called “heroes” then [122], why were they now treated as dispensable? In the words of one vaccinated worker: “In the [beginning] of the pandemic, we didn't get the vaccine, and we still had to work ... So now [they tell us] that we cannot work because of the same vaccine that we didn't get when we were working through the height of it?” [123] (pg. 665). Other studies showed “pushback” describing, for instance, how some HCWs had resisted the threat of mandatory vaccination and organized in-person protests and social media campaigns, successfully compelling the UK government to abandon the policy [124]. Interestingly, we found that pushback was not unique to high-income countries. For example, a study exploring support for vaccine mandates among HCWs in Nigeria reported that most participants felt that vaccination was an individual choice, and commented that more pressing medical problems had remained unaddressed in the country since the campaign, with one participant, a medical lab assistant, describing it as “a misplaced priority by the government... meant to please their international partners” [108] (pg. 10).

The body of data also revealed that “educational” efforts have generally failed, and that efforts to counter “misinformation” often backfired. For example, one study reported that mandates had not “significantly” impacted the first dose uptake among a sample of Canadian HCWs, “indicating a lack of significant change amongst those who decisively rejected vaccination” [117] (pg. 56). Another study asserted that for HCWs opposing vaccines—because, in the authors' views, they were misinformed—“information based messaging alone is likely to be ineffective” and may have led to a “backfire effect”, whereby individuals “become even more entrenched in their acceptance of misinformation” [87] (pg. 576). Authors also recognized that insisting on mandated vaccination had at times undermined public trust in social institutions. For example, one study participant wondered whether boosters were a “money grab” by pharmaceutical companies and were mandated by employers or governments for that reason, rather than for their alleged benefits [88] (pg. 701). Nevertheless, many authors still insisted that the policy of mandated vaccination enjoyed high support among HCWs, assuming, with no evidence, that support was indicated by the very high uptake found among HCWs [92].

## 5. Discussion

Across the body of data, authors appeared eager to provide evidence in support of the policy of universal vaccination for HCWs, mandatory if necessary, and tailored their research toward examining, understanding, and proposing a range of interventions to overcome all forms of resistance. For this reason, we concluded that the “problem” was represented to be the *targets* of these interventions, namely, dissenting HCWs who, allegedly for the greater good, and even for their *own* good, *had to be* vaccinated. It served this argument that vaccine uptake among HCWs was found to be high, higher than among the general public, which was interpreted as *acceptance* of the practice, missing the point that “uptake” may indicate not *acceptance* but *coercion*, if achieved through threats of undesirable consequences. Even authors who appeared more sympathetic and open to considering the experience of HCWs in their own terms entirely sidestepped the evidence informing these workers' resistance. This point was apparent from the fact that never were hesitancy, mistrust, or rejection of vaccination interpreted as reasonable responses to legitimate concerns about its safety, effectiveness, and necessity, or as a principled rejection of mandatory medical interventions more generally, but rather as peculiar features of workers' cognitions, attitudes, emotions, and behaviors that could be “managed” if only their trust were regained, or their will bent.

The single exception to this trend was mistrust when expressed by racialized HCWs, which was excused as resulting from historical or current collective experiences of racism, albeit not grounded on any *evidence* for distrusting *COVID-19* vaccination per se. It followed from this assumption that authors framed mistrust as “correctable” through “education” and improved “messaging” vis-à-vis racialized communities. However, many HCWs resisted. Others “chose” to be vaccinated, yet were not reported as having changed their beliefs, not changed and “vaccinate-or-terminate” policies appeared to be very unpopular regardless of vaccination status. In the end, studies still offered evidence that vaccinated *and* unvaccinated HCWs were negatively affected by their different stances toward vaccination, experiencing fractured relationships with peers, family members, and the larger community.

Several discursive mechanisms were deployed to achieve this dominant representation, salient among them logical *fallacies* [125], *doublespeak* [126], and *framing* [127]. One frequent logical fallacy was the use of *double standards* such that the representation of the views of supporters and opponents of vaccination mandates were treated differently for no legitimate reasons: the latter were dismissed as unwarranted, whereas the former were simply asserted to be based on science and good judgement, thus “confirming” the assumptions of study authors. This mechanism also involved *circular reasoning fallacies* in which assertions—e.g., that HCWs are “misinformed”—hinged on the very assumptions that needed to be proven—e.g., that their beliefs were “misinformation”. *Appeals to authority* were also widely used, such as the authority of the CDC, to counter decades of research on the protective role of natural immunity. Yet another fallacy was *appealing to emotions*, i.e., to the good nature of readers, likely to respond favorably to HCWs embracing a policy presented as necessary to protect patients and health systems, and conversely, unfavorable to those who did not. The fallacy of *special pleading* was applied to frame the concerns and mistrust expressed by racialized HCWs as legitimate, *while at the same time subtly dismissing them* as based on past, or contemporary, experiences of abuse, *yet not applicable to the case of COVID-19 policy*. The authors appeared to assume that readers would accept as “common sense” that racialized HCWs *should* trust authorities because *this time around* they were right. Finally, *ad hominem fallacies* were deployed to discredit challenges to the “scientific consensus”, which dispensed claims-makers from the need to provide any evidence.

*Doublespeak* was instrumental to presenting coercive medical interventions as “equitable”—allegedly benefiting the most vulnerable, even if unwilling, recipients—while the same speech act discursively dismissed the values of equity, diversity, and inclusivity, by promoting the inequitable exclusion of diverse points of view among HCWs. *Framing* in favor of the policy of mandatory vaccination was achieved by omitting contextual elements that would have allowed independent readers to fully evaluate authors' claims. Missing contexts included the dynamic nature of microbial virulence—founded on decades-old research into the complex relationship between hosts, their environments, and microbial agents [128],[129], the role of the social determinants of health in decreasing morbidity and mortality over close to 200 years, *before* the existence or mass implementation of any major medical intervention such as antibiotics or vaccines [130]–[132], the critical protective role afforded by natural immunity [45],[133], and the potential immunological impact of the psychological distress [134],[135] caused by COVID-19 countermeasures [136],[137].

Finally, the suffering of an unknown number of HCWs worldwide, and its ripple effect on families and communities, was either not mentioned or was justified as a scientifically and ethically informed response to the peculiarities of certain HCWs. These HCWs were assumed to be “hesitant” because they were at best “misinformed”—not “health literate” enough to understand the “scientific consensus”—or at worst “conspiratorial”, not because of the *substance* of their beliefs, which was never refuted, but because these HCWs disagreed with the views of recognized “epistemic authorities”, i.e., authorities approved by “experts” themselves [138]. Indeed, we have found these assumptions to recur in much of the expert literature on COVID-19 vaccination [139]–[142]. Meanwhile, the coercion built into the structure of this “our way or the highway” policy was ignored or justified on the grounds of its contribution to the “common good”, a discursive move that presented moral preferences in the guise of a mystifying medical expertise [143], while the tensions between the preferred policy and long-standing bioethical principles—informed consent, bodily autonomy, first do no harm, and so on—were not mentioned. Our finding is consistent with the study of elderly care homes in England cited earlier, in which researchers also identified—unsurprisingly, in our view—that an element of coercion was more effective in achieving vaccination goals in areas of higher unemployment, i.e., with fewer alternative options for workers refusing mandates [59]. It is worth noting once again that mandates also reduced staffing levels in that sector by an estimated 14,000 to 19,000 HCWs, with significant negative impact on patient care [59]. Notably, the fact that many dissenting HCWs, in addition to suffering personal and professional harms, were also barred from public spaces, and that many are still being kept away from jobs in the health sector, was entirely absent from discussions around the policy.

Our study has limitations, including its small sample size, its interpretive nature, and personal and professional biases. Another limitation is that our resources only allowed us to include English language articles, a criterion that may have introduced a language bias to our findings. Nevertheless, the country affiliation of authors was diverse enough to ensure that the problem representation we identified is shared by experts beyond the English-speaking world. Another limitation is the documentary nature of the data, as contacting the authors may have broadened our understanding of the problem representation we identified. On the other hand, these limitations are shared by other researchers, for instance, the authors of the literature we reviewed, whose work included linguistic and data type restrictions. To offset these limitations, we have presented detailed arguments and made our research process as transparent as possible so that other researchers can assess its quality and trustworthiness [144].

## 6. Conclusions

The question, however, remains: what explains the remarkable homogeneity of the dominant problem representation? We can only offer tentative answers, material and ideological. Material explanations may include the fact that while most authors declared not to have conflicts of interest, about 20% declared they did. Given the well-documented power that conflicts of interest exert, for instance, in the field of medical practice, policy, and research [145],[146], there is reason to believe that this influence also pervades researchers dedicated to investigating patterns of behavior within the healthcare labor force, especially when authors derive financial benefits from, or hold advisory roles with, vaccine manufacturers, pharmaceutical companies, and organizations whose mission *presupposes* that any degree of “vaccine scepticism” is a “problem” that requires “fixing” (see for instance [81]). Financial conflicts of interest, however, would not explain why the dominant problem representation prevailed even among authors who did not report these conflicts.

We have elaborated on this point elsewhere and restate here the thrust of our argument: we propose that the narratives of blame and shame against HCWs presenting a range of degrees of resistance to official policy in the health sector are not an isolated phenomenon. Indeed, fear-producing health narratives that draw from (pseudo) scientific ideas and real or imagined consensuses have been deployed throughout history. While their objects have shifted over time, they resemble one another in that they have effectively suppressed dissent and unrest, drawing from what has been dubbed a “cult of expertise” [147]. Selected examples include the fear of European Jews, blamed for the bubonic plague to manage social struggles in the late Middle Ages [148], fear of lepers, revived from biblical times in the late 19^th^ century to isolate immigrants from locals [149], fear of people of Chinese ancestry, who in the year 1900 were forcibly quarantined and mass inoculated with experimental vaccines upon the detection of cases of bubonic plague in San Francisco's Chinatown, to legitimize existing Sinophobic sentiment [150], and fear of “tramps,” blamed for smallpox in the early 20^th^ century [151] to redirect popular anger away from the dire living conditions that put the masses at risk of the disease and death blamed on the homeless [152].

We argue that in our era, COVID-19 is playing a functionally equivalent ideological role. Unlike in the past, the social identity of “the Other” under COVID-19 is multiply constituted, cutting across race/ethnicity, gender, class, and political ideology, yet unified, in that it threatens the global social order on health matters. This “Other” is projected by the establishment as challenging social spaces beyond health: thus, official documents warn about “misinformation” threatening global health as well as global finance, the survival of the planet, trust in political institutions, and the very nature of democracy, calling for “managing” or even “suppressing” dissenters by setting “safe” boundaries, physical and virtual, to permissible cognitions, attitudes, behaviors, and even speech acts (see for instance [153]–[157]). These boundaries have made the “unthinkable thinkable” [158], legitimizing a permanent “state of exception” [159] through state promoted “nudges” [160], and coercion if perceived as necessary to fight wars against yet-to-be-known, yet certain-to-happen, health threats [161].

Along the way, a system has been put in motion that discriminates against “Others” in multiple social spaces, like academia and the media, and, in the world of healthcare, “deviant” HCWs, even at risk of their livelihoods and lives, as compellingly displayed throughout the data. Importantly, this discriminatory system also violates fundamental bioethical principles, such as informed consent, i.e., the right to be fully and honestly informed about the risks and benefits of, and alternatives to, any medical intervention, to be offered the alternative to do nothing, and to be able to choose free from coercion. It is also at odds with the principles of equity, diversity, and inclusivity, held normatively in high esteem [162]–[165] by the same institutions that appear to subvert them in practice. A comprehensive evaluation of all relevant scientific, ethical, and legal aspects of the policy of mandated vaccination for HCWs is beyond the scope of this study, of our personal resources and capabilities, and of any single study. We trust, however, that our work can still contribute to a better-informed public debate around mandated vaccination and around COVID-19 policy more generally moving forward.


