# Supplementary material for: Is resistance to Covid-19 vaccination a “problem”? A critical policy inquiry of vaccine mandates for healthcare workers
Source: AIMS Public Health. 2024 Jun 12;11(3):688–714. doi: 10.3934/publichealth.2024035 (PMC11474332; doi:10.3934/publichealth.2024035)
Supplement: Supplementary file 1 [file publichealth-11-03-035-s001.pdf]

AIMS Public Health

AIMS Public Health, 11(3): 688–714.
DOI: 10.3934/publichealth.2024035
Received: 05 March 2024
Revised: 05 March 2024
Accepted: 07 May 2024
Published: 12 June 2024

https://www.aimspress.com/journal/aimsph

Research article

Is resistance to Covid-19 vaccination a "problem"? A critical policy inquiry of vaccine mandates for healthcare workers

Claudia Chaufan* **and Natalie Hemsing**

School of Health Policy and Management, York University, 4700 Keele St, Toronto, ON, M3J 1P3, Canada

* **Correspondance:** Email: cchaufan@yorku.ca.

Supplementary

Table 1. Included studies – basic characteristics.

ID	Reference	1st author affiliation	Date (year)	Country of Study	Study type	Study population
1.	Achat, Helen M, Joanne M Stubbs, and Rakhi Mittal. 2022. "Australian Healthcare Workers and COVID-19 Vaccination: Is Mandating Now or for Future Variants Necessary?" Australian and New Zealand Journal of Public Health 46 (1): 95. https://doi.org/10.1111/1753-6405.13191	Epidemiology and Health Analytics, Research and Education Network, Western Sydney Local Health District, New South Wales	2022	Australia	cross-sectional survey	hospital staff
2.	Badahdah, Abdallah M., Nawal Al Mahyijari, Ryan Badahdah, Furqan Al Lawati, and Faryal Khamis. 2022. "Attitudes of Physicians and Nurses in Oman Toward Mandatory COVID-19 Vaccination." Oman Medical Journal 37 (5): e429–e429. https://doi.org/10.5001/omj.2022.87	Department of Sociology and Rural Studies, School of Psychology, South Dakota State University, South Dakota	2022	Oman	cross-sectional survey	unvaccinated physicians and nurses
3.	Casey, Sharon M, Shana AB Burrowes, Taylor Hall, Sidney Dobbins, Mengyu Ma, Ruqiyya Bano, Christina Yarrington, Elissa M Schechter-Perkins, Christopher Garofalo, and Mari-Lynn Drainoni. 2022. "Healthcare Workers' Attitudes on Mandates, Incentives, and Strategies to Improve COVID-19 Vaccine Uptake: A Mixed Methods Study." *Human Vaccines & Immunotherapeutics* 18 (6): 2144048.	Department of Obstetrics and Gynecology, Boston University Chobanian and Avedisian School of Medicine, Boston, Massachusetts	2022	USA	mixed methods (survey & interviews)	health- care workers (all professions)
4.	Choi, Kristen, John Chang, Yi X. Luo, Bruno Lewin, Corrine Munoz-Plaza, David Bronstein, June Rondinelli, and Katia Bruxvoort. 2022. "'Still on the Fence': A Mixed Methods Investigation of COVID-19 Vaccine Confidence Among Health Care Providers." *Workplace Health & Safety* 70 (6): 285–97. https://doi.org/10.1177/21650799211049811.	University of California, Los Angeles	2022	USA	mixed methods (cross sectional survey & interviews)	physicians, advanced practice providers, nurses, and pharmacists
5.	Chrissian, Ara A, Udochukwu E Oyoyo, Pranjal Patel, W Lawrence Beeson, Lawrence K Loo, Shahriyar Tavakoli, and Alex Dubov. 2022. "Impact of COVID-19 Vaccine-Associated Side Effects on Health Care Worker Absenteeism and Future Booster Vaccination." *Vaccine* 40 (23): 3174–81.	Division of Pulmonary, Critical Care, Hyperbaric, Allergy, and Sleep Medicine, Loma Linda University, Loma Linda, CA,	2022	USA	cross-sectional survey	COVID-19-vaccinated staff in two healthcare systems
6.	De Graaf, Ysanne, Ber Oomen, Enrique Castro-Sanchéz, Jeannette Geelhoed, and Hubertus Johannes Maria Vrijhoef. 2023. "Nurses' Roles, Views and Knowledge Regarding Vaccines and Vaccination: A Pan-European Survey." *International Journal of Care Coordination* 26 (3–4): 129–36. https://doi.org/10.1177/20534345231207527.	Panaxea b.v., Den Bosch, The Netherlands	2023	Pan-European	cross-sectional survey	nurses

7.	Dennis, Amelia, Charlotte Robin, Leah Ffion Jones, and Holly Carter. 2022. "Exploring Vaccine Hesitancy in Care Home Employees in North West England: A Qualitative Study." *BMJ Open* 12 (5): e055239.	University of Bath, Bath, UK	2021	England	qualitative (interviews)	care home employees
8.	Déom, Noémie, Samantha Vanderslott, Patricia Kingori, and Sam Martin. 2023. "Online on the Frontline: A Longitudinal Social Media Analysis of UK Healthcare Workers' Attitudes to COVID-19 Vaccines Using the 5C Framework." *Social Science & Medicine* 339 (December): 116313. https://doi.org/10.1016/j.socscimed.2023.116313.	Department of Targeted Intervention, University College London, London, UK	2023	UK	qualitative (discourse analysis; Twitter network analysis)	UK healthcare workers
9.	Dietrich, Léna G, Alyssa Lüthy, Pia Lucas Ramanathan, Nadja Baldesberger, Andrea Buhl, Lisa Schmid Thurneysen, Lisa C Hug, L Suzanne Suggs, Camilla Speranza, and Benedikt M Huber. 2022. "Healthcare Professional and Professional Stakeholders' Perspectives on Vaccine Mandates in Switzerland: A Mixed-Methods Study." *Vaccine* 40 (51): 7397–7405.	University of Basel, Basel, Switzerland	2022	Switzerland	mixed methods (survey, interviews, focus groups)	healthcare providers, including physicians, pharmacists, nurses, midwives
10.	Digregorio, Marina, Pauline Van Ngoc, Simon Delogne, Eline Meyers, Ellen Deschepper, Els Duysburgh, Liselore De Rop, et al. 2022. "Vaccine Hesitancy towards the COVID-19 Vaccine in a Random National Sample of Belgian Nursing Home Staff Members." *Vaccines* 10 (4): 598. https://doi.org/10.3390/vaccines10040598	Research Unit of Primary Care and Health, Department of General Medicine, Faculty of Medicine, University of Liège, Belgium	2022	Belgium	cross-sectional survey	nursing home staff
11.	Dubov, Alex, Brian J. Distelberg, Jacinda C. Abdul-Mutakabbir, W. Lawrence Beeson, Lawrence K. Loo, Susanne B. Montgomery, Udochukwu E. Oyoyo, et al. 2021. "Predictors of COVID-19 Vaccine Acceptance and Hesitancy among Healthcare Workers in Southern California: Not Just 'Anti' vs. 'Pro' Vaccine." *Vaccines* 9 (12): 1428. https://doi.org/10.3390/vaccines9121428.	School of Behavioral Health, Loma Linda University, Loma Linda, CA	2021	USA	cross-sectional survey	nurses, physicians, other allied health professionals, and administrators
12.	Dzieciolowska, Stefania, Denis Hamel, Souleymane Gadio, Maude Dionne, Dominique Gagnon, Lucie Robitaille, Erin Cook, Isabelle Caron, Amina Talib, and Leighanne Parkes. 2021. "Covid-19 Vaccine Acceptance, Hesitancy, and Refusal among Canadian Healthcare Workers: A Multicenter Survey." *American Journal of Infection Control* 49 (9): 1152–57.	McGill University Faculty of Medicine, Montreal, Canada	2021	Canada	cross sectional survey	healthcare workers (all professions), including nurses, physicians, managers, administrative, technicians, allied health)

13.	Evans, Charlesnika T, Benjamin J DeYoung, Elizabeth L Gray, Amisha Wallia, Joyce Ho, Mercedes Carnethon, Teresa R Zembower, Lisa R Hirschhorn, and John T Wilkins. 2022. "Coronavirus Disease 2019 (COVID-19) Vaccine Intentions and Uptake in a Tertiary-Care Healthcare System: A Longitudinal Study." *Infection Control & Hospital Epidemiology* 43 (12): 1806–12.	Department of Preventive Medicine, Feinberg School of Medicine, Northwestern University, Chicago, Illinois,	2022	USA	prospective cohort study	healthcare workers in Chicago (including physicians, registered nurses, administration)
14.	Fadda, Marta, Kleona Bezani, Rebecca Amati, Maddalena Fiordelli, Luca Crivelli, Emiliano Albanese, L Suzanne Suggs, and Maria Caiata-Zufferey. 2022. "Decision-Making on COVID-19 Vaccination: A Qualitative Study among Health Care and Social Workers Caring for Vulnerable Individuals." *SSM-Qualitative Research in Health* 2: 100181.	Institute of Public Health, Universita della Svizzera italiana, Lugano, Switzerland	2022	Switzerland	qualitative	staff employed in nursing homes and institutes for people with disabilities
15.	Ford, Abigail, Penny Heidke, Chanchal Kurup, Lisa Wirihana, Jeremy Kroll, and Pauline Calleja. 2023. "Factors Influencing Australian Nursing and Midwifery Students COVID-19 Vaccination Intentions." *Vaccine: X* 14 (August): 100352. https://doi.org/10.1016/j.jvacx.2023.100352.	School of Nursing, Midwifery and Social Sciences, CQUniversity Australia	2023	Australia	cross sectional mixed methods study	nursing and midwifery students
16.	Heyerdahl, Leonardo W, Stef Dielen, Hélène Dodion, Carla Van Riet, ToTran Nguyen, Clarissa Simas, Lise Boey, Tarun Kattumana, Nico Vandaele, and Heidi J Larson. 2023. "Strategic Silences, Eroded Trust: The Impact of Divergent COVID-19 Vaccine Sentiments on Healthcare Workers' Relations with Peers and Patients." *Vaccine* 41 (4): 883–91.	Department of Global Health, Anthropology and Ecology of Disease Emergence Unit, Institut Pasteur, Paris, France	2023	Belgium	qualitative	healthcare workers and students (including nurses, doctors, specialists, dentists, psychologists, ambulance drivers)
17.	Hoffman, Beth L, Cassandra L Boness, Kar-Hai Chu, Riley Wolynn, Larissa Sallowicz, Dino Mintas, Antoine B Douaihy, Elizabeth M Felter, and Jaime E Sidani. 2022. "COVID-19 Vaccine Hesitancy, Acceptance, and Promotion Among Healthcare Workers: A Mixed-Methods Analysis." *Journal of Community Health* 47 (5): 750–58.	Department of Behavioral and Community Health Sciences, University of Pittsburgh School of Public Health	2022	USA	mixed methods (survey, Twitter analysis)	all employees at a large psychiatric hospital
18.	Hubble, Michael W, Ginny K Renkiewicz, Sandy Hunter, and Randy D Kearns. 2022. "Predictors of COVID-19 Vaccination Among EMS Personnel." *Western Journal of Emergency Medicine* 23 (4): 570.	Wake Technical Community College, Department of Emergency Medical Science, Raleigh, North Carolina	2022	USA	cross-sectional survey	Emergency Medical Services (EMS) professionals

19.	Iliyasu, Zubairu, Rayyan M Garba, Mansur A Aliyu, Auwalu U Gajida, Taiwo G Amole, Amina A Umar, Hadiza M Abdullahi, Fatimah I Tsiga-Ahmed, Aminatu A Kwaku, and Meira S Kowalski. 2022. "'I Would Rather Take the Vaccine Than Undergo Weekly Testing': Correlates of Health Workers' Support for COVID-19 Vaccine Mandates." *International Journal of Environmental Research and Public Health* 19 (21): 13937.	Department of Community Medicine, Bayero University, Kano, Nigeria	2022	Nigeria	mixed methods (survey and interviews)	clinical and non-clinical staff at a tertiary hospital
20.	Iwu, Chinedu Anthony, Pius Ositadinma, Victor Chibiko, Ugochukwu Madubueze, Kenechi Uwakwe, and Uche Oluoha. 2022. "Prevalence and Predictors of COVID-19 Vaccine Hesitancy among Health Care Workers in Tertiary Health Care Institutions in a Developing Country: A Cross-Sectional Analytical Study." Edited by Francesco Chirico. *Advances in Public Health* 2022 (March): 1–9. https://doi.org/10.1155/2022/7299092.	Community Medicine Department, Imo State University, Imo State University Teaching Hospital, Owerri, Nigeria	2022	Nigeria	cross-sectional survey	healthcare workers in tertiary health care institutions
21.	Klugar, Miloslav, Abanoub Riad, Lekshmi Mohanan, and Andrea Pokorná. 2021. "COVID-19 Vaccine Booster Hesitancy (VBH) of Healthcare Workers in Czechia: National Cross-Sectional Study." *Vaccines* 9 (12): 1437. https://doi.org/10.3390/vaccines9121437.	Czech National Centre for Evidence-Based Healthcare and Knowledge Translation, Faculty of Medicine, Institute of Biostatistics and Analyses, Masaryk University, Czech Republic	2021	Czech Republic	cross-sectional survey	medical professionals and allied health professionals
22.	Lee, James T, S Sean Hu, Tianyi Zhou, Kimberly E Bonner, Jennifer L Kriss, Elisabeth Wilhelm, Rosalind J Carter, Carissa Holmes, Marie A de Perio, and Peng-jun Lu. 2022. "Employer Requirements and COVID-19 Vaccination and Attitudes among Healthcare Personnel in the US: Findings from National Immunization Survey Adult COVID Module, August–September 2021." *Vaccine* 40 (51): 7476–82.	Centers for Disease Control (CDC) COVID-19 Response	2022	USA	cross-sectional survey	employed in healthcare setting (e.g., hospital, doctor, dentist or mental health specialist office, outpatient facility, long-term care, home healthcare, pharmacy, medical laboratory)
23.	Lohiniva, Anna-Leena, Idil Hussein, Jaana-Marija Lehtinen, Jonas Sivelä, Suvi Hyökki, Hanna Nohynek, Pekka Nuorti, and Outi Lyytikäinen. 2023. "Qualitative Insights into Vaccine Uptake of Nursing Staff in Long-Term Care Facilities in Finland." *Vaccines* 11 (3): 530.	Finnish Institute for Health and Welfare, Mannerheimintie	2023	Finland	qualitative (interviews)	unvaccinated nursing staff in long-term care facilities

24.	Lucia, Victoria C, Arati Kelekar, and Nelia M Afonso. 2021. "COVID-19 Vaccine Hesitancy among Medical Students." *Journal of Public Health* 43 (3): 445–49. https://doi.org/10.1093/pubmed/fdaa230.	Foundational Medical Studies, Oakland University William Beaumont School of Medicine, Rochester, MI	2020	USA	cross-sectional survey	medical students
25.	Moudatsou, Maria, Areti Stavropoulou, Michael Rovithis, and Sofia Koukouli. 2023. "Views and Challenges of COVID-19 Vaccination in the Primary Health Care Sector. A Qualitative Study." *Vaccines* 11 (4): 803.	Social Work Department, School of Health Sciences, Hellenic Mediterranean University, Heraklion, Greece	2023	Greece	qualitative	health professionals working in primary health and social care services
26.	Oberleitner, Lindsay MS, Victoria C Lucia, Mark C Navin, Melissa Ozdych, Nelia M. Afonso, Richard H Kennedy, Hans Keil, Lawrence Wu, and Trini A Mathew. 2022. "COVID-19 Vaccination Concerns and Reasons for Acceptance Among US Health Care Personnel." *Public Health Reports* 137 (6): 1227–34.	William Beaumont School of Medicine, Oakland University, Rochester, MI	2022	USA	cross-sectional survey	employees of a large health system - including clinical or nonclinical care
27.	Okpani, Arnold I., Karen Lockhart, Jennifer M. Grant, Stephen Barker, Jocelyn A. Srigley, and Annalee Yassi. 2023. "Vaccination, Time Lost from Work, and COVID-19 Infections: A Canadian Healthcare Worker Retrospective Cohort Study." *Frontiers in Public Health* 11 (August): 1214093. https://doi.org/10.3389/fpubh.2023.1214093.	School of Population and Public Health, University of British Columbia, Vancouver, BC.	2023	Canada	retrospective cohort	staff providing all laboratory, hospital, and long-term care services
28.	Plummer, Elizabeth, and William F. Wempe. 2023. "Evidence on the Effects of the Federal COVID-19 Vaccine Mandate on Nursing Home Staffing Levels." *Journal of the American Medical Directors Association* 24 (4): 451–58. https://doi.org/10.1016/j.jamda.2022.12.024.	TCU Neeley School of Business/TCU School of Medicine, Texas Christian University, Fort Worth, TX	2023	USA	cross sectional study	nursing home staff
29.	Poyiadji, Neo, Alexander Tassopoulos, Daniel T Myers, Lauren Wolf, and Brent Griffith. 2022. "COVID-19 Vaccine Mandates: Impact on Radiology Department Operations and Mitigation Strategies." *Journal of the American College of Radiology* 19 (3): 437–45.	Department of Diagnostic Radiology, Henry Ford Hospital, Detroit, Michigan	2022	USA	retrospective cohort study	employees in radiology department
30.	Ritter, Ashley Z., Jennifer Kelly, Robert M. Kent, Pamela Howard, Robert Theil, Priya Cavanaugh, James Hollingsworth, Joseph S. Duffey, Mary Schuler, and Mary D. Naylor. "Implementation of a coronavirus disease 2019 vaccination condition of employment in a community nursing home." *Journal of the American Medical Directors Association* 22, no. 10 (2021): 1998-2002.	New Courtland Center for Transitions and Health, University of Pennsylvania School of Nursing, Philadelphia, PA	2021	USA	case study	nursing home staff

31.	Russell, David, Nicole Onorato, Alexis Stern, Sasha Vergez, Mia Oberlink, Matthew Luebke, Penny H Feldman, Margaret V McDonald, and Madeline R Sterling. 2023. "A Qualitative Study of Home Health Aides' Perspectives towards COVID-19 Vaccination." *Journal of Applied Gerontology* 42 (4): 660–69.	Department of Sociology, Appalachian State University, Boone, NC	2023	USA	qualitative (interviews)	home health aides
32.	Shaw, Jana, Samantha Hanley, Telisa Stewart, Daniel A Salmon, Christine Ortiz, Paula M Trief, Elizabeth Asiago Reddy, Christopher P Morley, Stephen J Thomas, and Kathryn B Anderson. 2022. "Healthcare Personnel (HCP) Attitudes About Coronavirus Disease 2019 (COVID-19) Vaccination After Emergency Use Authorization." *Clinical Infectious Diseases* 75 (1): e814–21. https://doi.org/10.1093/cid/ciab731.	Department of Pediatrics, Division of Infectious Diseases, State University of New York (SUNY) Upstate Medical University, Syracuse, New York,	2022	USA	cross sectional survey	clinical and nonclinical healthcare staff, researchers, and trainees
33.	Singh, Arvind Kumar, Rashmi Kumari, Shikhar Singh, Sunil Dutt Kandpal, and Amit Kaushik. 2021. "The Dilemma of COVID-19 Vaccination among Health Care Workers (HCWs) of Uttar Pradesh." *Indian Journal of Community Health* 33 (2): 319–24. https://doi.org/10.47203/IJCH.2021.v33i02.017.	Associate Professor, Department of Community Medicine, Dr. Ram Manohar Lohia Institute of Medical Sciences, Lucknow	2021	India	cross sectional survey	doctors and paramedical workers
34.	Syme, Maggie L., Natalia Gouskova, and Sarah D. Berry. 2022. "COVID-19 Vaccine Uptake Among Nursing Home Staff via Statewide Policy: The Mississippi Vaccinate or Test Out Policy." *American Journal of Public Health* 112 (5): 762–65. https://doi.org/10.2105/AJPH.2022.306800.	Hinda and Arthur Marcus Institute for Aging Research, Hebrew Senior Life, Boston, MA	2022	USA	cross sectional study	nursing home staff
35.	Thaivalappil, Abhinand, Ian Young, Melissa MacKay, David L Pearl, and Andrew Papadopoulos. 2022. "A Qualitative Study Exploring Healthcare Providers' and Trainees' Barriers to COVID-19 and Influenza Vaccine Uptake." *Health Psychology and Behavioral Medicine* 10 (1): 695–712.	Department of Population Medicine, University of Guelph, Guelph, ON	2022	Canada	qualitative (interviews)	active or retired healthcare providers or healthcare students
36.	Tylec, Aneta, Mariola Janiszewska, Krzysztof Siejko, and Katarzyna Kucharska. 2023. "Determinants of the Decision to Be Vaccinated against COVID-19 as Exemplified by Employees of a Long-Term Health Care Centre." *Journal of Public Health* 45 (1): 237–44. https://doi.org/10.1093/pubmed/fdab395.	Department of Psychiatry, Medical University of Lublin, Lublin, Poland	2021	Poland	cross sectional survey	employees of long-term healthcare centre

37.	Ulrich, Angela K, Grace K Pankratz, Bruno Bohn, Stephanie Yendell, Timothy J Beebe, Craig W Hedberg, and Ryan T Demmer. 2022. "COVID-19 Vaccine Confidence and Reasons for Vaccination among Health Care Workers and Household Members." *Vaccine* 40 (41): 5856–59.	Center for Infectious Disease Research and Policy, University of Minnesota, Minneapolis	2022	USA	cross-sectional survey	healthcare workers (physician, nurse, nurse practitioner, laboratory technician, administration, paramedic or EMT) and their families
38.	Viskupič, Filip, and David L. Wiltse. 2023. "Partisan Self-Identification Predicts Attitudes of South Dakota Nurses toward COVID-19 Vaccine Mandate for Healthcare Workers." *Health Policy and Technology* 12 (3): 100777. https://doi.org/10.1016/j.hlpt.2023.100777.	School of American and Global Studies, South Dakota State University	2023	USA	cross-sectional survey	nurses
39.	Wirpsa, M.J., P. Galchutt, C.S. Price, B. Schaefer, C. Szilagyi, and P.K. Palmer. 2023. "Mandatory COVID-19 Vaccination for Healthcare Workers: The Experience of Chaplains Evaluating Religious Accommodation Requests from Coworkers." *Social Science & Medicine* 332 (September): 116103. https://doi.org/10.1016/j.socscimed.2023.116103.	Northwestern Memorial Hospital, Chicago, IL	2023	USA	mixed methods (survey, interviews)	healthcare chaplains
40.	Woodhead, Charlotte, Juliana Onwumere, Rebecca Rhead, Monalisa Bora-White, Zoe Chui, Naomi Clifford, Luke Connor, Cerisse Gunasinghe, Hannah Harwood, and Paula Meriez. 2022. "Race, Ethnicity and COVID-19 Vaccination: A Qualitative Study of UK Healthcare Staff." *Ethnicity & Health* 27 (7): 1555–74.	Department of Psychological Medicine, Institute of Psychiatry, Psychology and Neuroscience, King's College London	2022	UK	qualitative (interviews)	student nurses, healthcare assistants, and qualified nurses within the NHS (UK)
41.	Yassi, Annalee, Stephen Barker, Karen Lockhart, Deanne Taylor, Devin Harris, Harsh Hundal, Jennifer M Grant, Arnold Ikedichi Okpan, Sue Pollock, and Stacy Sprague. 2023. "Urban-Rural Divide in COVID-19 Infection and Vaccination Rates in Healthcare Workers in British Columbia, Canada." *Canadian Journal of Rural Medicine* 28 (2): 47–58.	School of Population and Public Health, University of British Columbia, Vancouver, British Columbia	2023	Canada	retrospective cohort analysis	employees of health authorities (Interior Health; Vancouver Coastal Health); therefore, physicians were not included

Table 2. Included studies: Funding Sources & Conflicts of Interest (COI).

ID	**Funding**<br>**Declared (yes/no) / Present (yes/no)**	**COI**<br>**Declared (yes/no) / Present (yes/no)**
1.	No	No
2.	Yes (no funding)	Yes (no COI)
3.	Yes - Grant from Boston University School of Medicine Clinical and Translational Science Institute	Yes (no COI)
4.	Yes - Grant from Care Improvement Research Team (CIRT), Kaiser Permanente Southern California	Yes - No COI related to the contents of the article. Y.L. discloses funding unrelated to this study from GlaxoSmithKline, Novavax, Seqirus, Moderna. K.B. discloses funding unrelated to this study from GlaxoSmithKline, Seqirus, Gilead, Dynavax, Pfizer, and Moderna)
5.	Yes (none)	Yes (no COI)
6.	Yes - Vaccines Europe	Yes - HV is the Editor-in-Chief of the International Journal of Care Coordination; did not handle editorial processing of this article)
7.	Yes (none)	Yes (no COI)
8.	Yes - Grant from John Fell Fund	No
9.	Yes - Swiss National Science Foundation	Yes (no COI)
10.	Yes - Belgian Scientific Institute of Public Health (SCIENSANO)	Yes (no COI)
11.	Yes - Center for HIV Identification, Prevention, and Treatment Services	Yes (no COI)
12.	Yes- Centre intègre universitaire de santé et services sociaux centre-ouest-de- l'ile-de-Montréal (CIUSSS) and Institut national de sante publique du Québec (INSPQ))	Yes - Dr Longtin reported receiving research grants from Merck, Becton Dickinson and Gojo)
13.	Yes - Northwestern University Clinical and Translational Sciences Institute and the Northwestern Memorial Foundation	Yes (no COI)
14.	No	Yes - L. Suzanne Suggs reports a relationship with MSD European Vaccines that includes board membership
15.	Yes - CQ University Internal Research Grants	Yes (no COI)
16.	Yes - Vaccine Confidence Fund	Yes - Koen Peeters Grietens reports financial support from Vaccine Confidence Fund. Heidi J Larson reports a relationship with Ellen MacArthur Foundation that includes funding grants. Heidi J Larson reports a relationship with Johnson & Johnson that includes funding grants. Heidi J Larson reports a relationship with UNICEF that includes funding grants. Koen Peeters Grieten reports a relationship with Research Foundation Flanders that includes funding grants
17.	Yes - Richard King Mellon Foundation	Yes (no COI)
18.	Yes (none)	Yes (no COI)
19.	Yes (none)	Yes (no COI)
20.	No	Yes (no COI)
21.	Yes - Grant from Masaryk University. The work of M.K., A.R. and A.P. was supported by the INTER-EXCELLENCE grant number LTC20031 "Towards an International Network for Evidence-based Research in Clinical Health Research in the Czech Republic"	Yes (no COI)
22.	No	Yes (no COI)
23.	Yes (none)	Yes (no COI)
24.	No	Yes (no COI)
25.	Yes - Special Account for Research Funds of the Hellenic Mediterranean University	Yes (no COI)
26.	Yes (none)	Yes (no COI)
27.	No	Yes (no COI)
28.	Yes (none)	Yes (no COI)
29.	No	Yes (no COI)
30.	No	Yes (no COI)

31.	Yes - Grant from the Altman Foundation	Yes (no COI)
32.	Yes (none)	Yes - J. S. reports payment/honoraria for serving on a Speaker Bureau for Pfizer. S. J. T. reports grants/support from Department of Defense (DOD), National Institutes of Health (NIH) (National Institutes of Allergy and Infectious Diseases [NIAID], National Institute on Drug Abuse [NIDA]), Pfizer, Janssen, and Merck (only Pfizer contract with University as clinical trial site relates to the content of the manuscript [COVID vaccine trial]); consulting fees from Takeda, Pfizer, Janssen, Merck, Sanofi, Clover Pharm, Icosavaxx, and Tremeau (consulting to Pfizer for COVID vaccine); payment for serving as expert witness to inform court on infectious disease issues related to med mal cases; travel support if travel was related to consulting activities, the following patents: zika vaccine (issued); flavivirus vaccine (issued); chi- kungunya (under review); participation in Data and Safety Monitoring Board (DSMB)/Advisory Boards for Takeda, Merck, Sanofi, Janssen, PATH, Icossavax, and Moderna (Icosavaxx related to COVID vaccine trial); the fol lowing leadership/fiduciary roles: board of directors (BoD) – Byrne Dairy; board of managers – Skaneateles YMCA; BoD – CNY Lyme Alliance; BoD – Skaneateles Music Festival; Co-founder, Phairify Inc; Cofounder, Cormac life sciences; stock/stock options/equity for scientific advisory board membership from PrimeVax; all outside the submitted work. C. P. M. reports grants/support from New York State Health Foundation, Health Resources and Services Administration (HRSA), and HealthResearch, Inc (New York State Department of Health [NYSDOH]); payments/honoraria for HRSA Grant Review, SUNY Downstate Grant Review, and STFM Journal Editorship; travel support from North American Primary Care Research Group (NAPCRG) (travel to AFMAC advisory meeting); the following leadership/fiduciary roles: Member, Society of Teachers of Family Medicine (STFM), and NAPCRG representative, Academic Family Medicine Advocacy Committee; Editor, PRiMER journal (STFM); all outside the submitted work. D. A. S. reports consulting and/or research support from Jensen and Merck, outside the submitted work. K. A. reports consulting fees for Steering committee member, chikungunya vaccines from Emergent BioSolutions and chair, peer review from CDC, Dengue Branch; DSMB member for AstraZeneca, and data adjudication committee for Clover pharmaceuticals; councilor for Global Health subcommittee, ASTMH. All other authors report no potential conflicts. All authors have submitted the ICMJE Form for Disclosure of Potential Conflicts of Interest. Conflicts that the editors consider relevant to the content of the manuscript have been disclosed.
33.	Yes (none)	Yes (no COI)
34.	Yes - Grant from the National Institute on Aging)	Yes (no COI)
35.	No	No
36.	Yes - Ontario Graduate Scholarship; the University of Guelph's Ontario Veterinary College Scholarship	Yes (no COI)
37.	Yes - Minnesota Department of Health Contract	Yes - All authors reported financial support from Minnesota Department of Health and the Centers for Disease Control and Prevention.
38.	Yes - Supported by South Dakota State University	Yes (no COI)
39.	Yes - Transforming Chaplaincy at Rush University Health Center	Yes (no COI)
40.	Yes - Economic and Social Research Council	Yes (no COI)
41.	Yes - Canadian Institutes of Health Research (CIHR) under grant (VS1-175519), "Protecting healthcare workers from COVID-19: a comparative contextualized analysis". AY also received funding from the Canada Research Council as a CRC Chairholder	Yes (no COI)

Table 3. Summary of selected characteristics of included studies.

**Characteristic**	**No.; %**
**Year of Publication**	
2020	1/41; 2%
2021	21/41; 51%
2022	7/41; 17%
2023	12/41; 29%
**Country of Study**	
United States of America (USA)	19/41; 46%
Canada	4/41; 10%
United Kingdom	3/41; 7%
Australia	2/41; 5%
Belgium	2/41; 5%
Switzerland	2/41; 5%
Nigeria	2/41; 5%
Oman	1/41; 2%
Czech Republic	1/41; 2%
Finland	1/4; 2%
Greece	1/4; 2%
India	1/4; 2%
Poland	1/4; 2%
Pan-European	1/4; 2%
**Study type**	
Quantitative (observational)	25/41; 61%
Qualitative	9/41; 22%
Mixed methods	7/41; 17%
**Study Population**	
Nursing home and home care employees	9/41; 22%
Employees of particular medical department, hospital, or health system	8/41; 20%
Health care workers of all professions (including non-clinical, researchers, support staff)	6/41; 15%
8Patient care providers – including select multiple groups (e.g., physicians, nurses, EMT)	5/41/; 12%
Patient care providers and administrators	3/41; 7%
Nurses	3/41; 7%
Students (medical, nursing, midwifery)	2/41; 5%

Medical students and healthcare providers	2/41; 5%
Patient care and laboratory services	1/41; 2%
EMS professionals	1/41; 2%
Healthcare chaplains	1/41; 2%
**Funding Declared / Present**	
Yes	32/41; 78%
Yes	22/32; 69%
**Conflict of Interest Declared / Present**	
Yes	38/41; 82%
Yes	7/38; 18%

AIMS Press

© 2024 the Author(s), licensee AIMS Press. This is an open access article distributed under the terms of the Creative Commons Attribution License (https://creativecommons.org/licenses/by/4.0)
